# Evaluating the impact of early identification of asymptomatic brain metastases in metastatic renal cell carcinoma

**DOI:** 10.1002/cnr2.1763

**Published:** 2022-12-14

**Authors:** Ambica Parmar, Sunita Ghosh, Arjun Sahgal, Aly‐Khan A. Lalani, Aaron R. Hansen, M. Neil Reaume, Lori Wood, Naveen S. Basappa, Daniel Y. C. Heng, Jeffrey Graham, Christian Kollmannsberger, Denis Soulières, Rodney H. Breau, Simon Tanguay, Anil Kapoor, Frédéric Pouliot, Georg A. Bjarnason

**Affiliations:** ^1^ Odette Cancer Centre Sunnybrook Health Sciences Centre Toronto Ontario Canada; ^2^ Cross Cancer Institute University of Alberta Edmonton Alberta Canada; ^3^ Juravinski Cancer Centre McMaster University Hamilton Ontario Canada; ^4^ Princess Margaret Cancer Centre University Health Network Toronto Ontario Canada; ^5^ Ottawa Hospital Cancer Centre Ottawa Ontario Canada; ^6^ Dalhousie University Halifax Nova Scotia Canada; ^7^ Tom Baker Cancer Centre University of Calgary Calgary Alberta Canada; ^8^ Unversity of Manitoba Winnipeg Manitoba Canada; ^9^ BC Cancer – Vancouver Centre Vancouver British Columbia Canada; ^10^ Centre Hospitalier de l'Université de Montréal Montréal QC Canada; ^11^ McGill University Montréal QC Canada; ^12^ Cancer Research Center Centre Hospitalier Universitaire de Québec – Université Laval Québec City QC Canada

**Keywords:** brain metastases, imaging, radiation therapy, renal cell carcinoma, surveillance

## Abstract

**Background:**

Brain metastases (BM) in metastatic renal cell carcinoma (mRCC) have been reported to be present in up to 25% of patients diagnosed with mRCC. There is limited published literature evaluating the role of routine intra‐cranial imaging for the screening of asymptomatic BM in mRCC.

**Aims:**

To evaluate the potential utility of routine intra‐cranial imaging, a retrospective cohort study was conducted to characterize the outcomes of mRCC patients who presented with asymptomatic BM, as compared to symptomatic BM.

**Methods and Results:**

The Canadian Kidney Cancer Information System (CKCis) database was used to identify mRCC patients diagnosed with BM. This cohort was divided into two groups based on the presence or absence of BM symptoms. Details regarding patient demographics, disease characteristics, systemic treatments, BM characteristics and survival outcomes were extracted. Statistical analysis was through chi‐square tests, analysis of variance, and Kaplan–Meier method to characterize survival outcomes. A *p*‐value of <0.05 was considered statistically significant for all analyses. A total of 267 mRCC patients with BM were identified of which 106 (40%) presented with asymptomatic disease. The majority of patients presented with multiple (i.e., >1) BM (75%) with no significant differences noted in number of BM or BM‐directed therapy received in symptomatic, as compared to asymptomatic BM patients. Median [95% confidence interval (CI)] overall survival (OS) from mRCC diagnosis was 42 months (95% CI: 32–62) for patients with asymptomatic BM, and 39 months (95% CI: 29–48) with symptomatic BM (p = 0.10). OS from time of BM diagnosis was 28 months (95% CI: 18–42) for the asymptomatic BM group, as compared to 13 months (95% CI: 10–21) in the symptomatic BM group (p = 0.04).

**Conclusions:**

Given a substantial proportion of patients may present with asymptomatic BM, limiting intra‐cranial imaging to patients with symptomatic BM, may be associated with a missed opportunity for timely diagnosis and treatment. The utility of routine intra‐cranial imaging in patients with renal cell carcinoma, warrants further prospective evaluation.

## 1 INTRODUCTION

Brain metastases (BM) in metastatic renal cell carcinoma (mRCC) have a reported incidence that ranges between 5% and 25%.^1^, ^2^ Until recently, national and international consensus guidelines have not recommended the routine use of intra‐cranial imaging to screen for BM in patients with renal cell carcinoma (RCC) in the absence of symptoms.^3^, ^4^, ^5^ The more recent 2022 European Association of Urology Guidelines on Renal Cell Carcinoma similarly do not recommend routine brain imaging for patients with RCC, but suggest offering brain imaging for patients with mRCC.^6^ It has been estimated that up to 30% of mRCC patients with BM are asymptomatic.^2^, ^7^ As such, restricting routine intra‐cranial imaging to symptomatic mRCC patients carries a risk of delays in diagnosis and subsequent treatment of BM.

Historically, the development of BM portended a poor prognosis with an expected survival of less than 1‐year.^2^ Systemic therapy for mRCC has been ineffective in providing adequate intra‐cranial disease control.^8^, ^9^ Neurosurgical resection is an effective option but is limited to solitary BM in accessible locations.^10^ Advances in modern radiation techniques have led to significant improvements in intra‐cranial disease control with the use of stereotactic radiosurgery (SRS) as compared to whole brain radiotherapy (WBRT), with SRS associated with higher rates of local control and lower rates of neurocognitive decline and deterioration in quality‐of‐life.^11^, ^12^ Specifically in mRCC patients with BM, SRS has been associated with high intra‐cranial disease control rates and manageable toxicities.^13^, ^14^, ^15^ However, the most effective application of SRS is in the setting of a low volume of intra‐cranial disease, with a limited number of small BM.^12^, ^14^ As such, earlier identification of BM may facilitate the timely application of more effective local therapies, such as SRS or neurosurgical intervention, to improve intra‐cranial disease control and maintain patient functional outcomes.

To date, there is a paucity of published literature evaluating the role of routine intra‐cranial imaging for the screening of asymptomatic BM in mRCC.^7^ As such, using a Canadian database, we conducted a retrospective cohort study to evaluate and compare the outcomes of mRCC patients who presented with asymptomatic as compared to symptomatic BM.

## 2 METHODS

### 2.1 Study population

The Canadian Kidney Cancer Information System (CKCis) database was used to identify patients with the following eligibility criteria for inclusion in our study: adult (age ≥ 18 years), diagnosis of mRCC and a diagnosis of BM between January 1, 2011 and December 31, 2018. Patients were ineligible for inclusion if they did not have a diagnosis of BM. The CKCis database is a national Canadian database that has been collecting information on all RCC patients across 16 participating Canadian centers since 2011 and has been shown to be representative of the Canadian population.^16^ Research ethics approval has been granted for all CKCis participating centers. In the CKCis database, we identified all patients with mRCC who were reported to have BM as a site of metastasis. For each patient identified to have BM, symptoms at the time of BM diagnosis are recorded in the CKCis database. Using these data, study investigators divided the cohort into two groups based on the presence (i.e., symptomatic) or absence (i.e., asymptomatic) of BM symptoms. Specifically, any recorded symptoms of headache, seizure or focal neurological deficits were coded as symptomatic BM. Although alternative symptoms, such as nausea, vomiting or fatigue, can also be present in patients with BM, given these are non‐specific and accordingly, potentially attributable to the diagnosis of mRCC or systemic treatment, we did not include these in our list of symptoms to identify symptomatic BM.

### 2.2 Measures and outcomes

For all included patients, the following data were collected: (a) baseline patient demographics (including age, sex); (b) disease characteristics (including histology, prognostic risk, as assessed by the International Metastatic Renal Cell Carcinoma Database Consortium (IMDC) prognostic model, prior nephrectomy status, sites of metastasis at time of BM diagnosis); (c) characteristics of BM (including number of BM, receipt of local therapy); and (d) survival outcomes.

### 2.3 Statistical analysis

All analysis was performed using the statistical software SAS version 9.3 (SAS Institute Inc., Cary, NC). All data were analyzed descriptively with frequencies and proportions for categorical data, means and standard deviations (SD) for normally distributed continuous variables, and medians and interquartile ranges (IQR) for non‐parametric continuous data. Proportions were compared using chi‐square tests. Means of continuous variables between two or more groups were compared using one‐way analysis of variance (ANOVA). The Kaplan–Meier (KM) method was utilized to estimate the median overall survival (OS) and the corresponding 95% confidence intervals (CI). Kaplan–Meier methods were also used to generate survival curves and log‐rank statistics were used to compare two or more OS survival curves. Overall survival was calculated both from the time of mRCC diagnosis and from the time of BM diagnosis (based on date of intra‐cranial imaging confirmation of BM) to the date of death or last date of documented follow‐up. Cox proportional hazards models were used to perform univariate and multivariable analyses to identify factors associated with OS from the time of mRCC diagnosis and presented as hazard ratios (HR) with corresponding 95% CI. A p‐value of <0.05 was used as the criteria for statistical significance.

## 3 RESULTS

### 3.1 Study cohort

From a total of 2889 mRCC patients captured in the CKCis database between 2011 and 2018, 267 patients (9.2%) were diagnosed with BM. Of these, 106 (40%) were diagnosed with asymptomatic BM and 161 (60%) were diagnosed with symptomatic BM.

Across the two groups of patients, no significant differences were noted with respect to disease characteristics (Table 1). Supplemental Figure 1 summarizes the sites of metastases at the time of diagnosis of BM across both groups. The majority of patients were found to have concomitant lung metastases with no significant differences between the two groups (asymptomatic: 77% vs. symptomatic: 75%, *p* = 0.60). Among the asymptomatic BM and symptomatic BM groups, 10 (9%) and 25 (15%), respectively, were found to have isolated BM with no other metastatic sites.

**TABLE 1:** Characteristics of cohort

Characteristic	Asymptomatic (*N* = 106)	Symptomatic^a^ (*N* = 161)	*p*‐value
Age (median (IQR); years)	62 (56–68)	61 (56–69)	0.90
> 65 years (%)	39 (37)	58 (36)	
Male sex (%)	81 (76)	119 (74)	0.64
Histology^b^ (%)			0.25
Clear‐cell	81/102 (79)	134/156 (86)	
Papillary	1/102 (1)	4/156 (3)	
Chromophobe	2/102 (2)	1/156 (1)	
Other	18/102 (18)	18/156 (12)	
Prior nephrectomy (%)	80 (75)	133 (83)	0.18
IMDC prognostic risk^b^ (%)			0.37
Favorable	12/68 (18)	10/98 (10)	
Intermediate	36/68 (53)	58/98 (59)	
Poor	20/68 (29)	30/98 (31)	
*Systemic therapy* ^c^ (%)			
First‐line	91 (86)	124 (77)	0.08
Sunitinib	72 (79)	90 (73)	0.27
Pazopanib	11 (12)	20 (16)	0.41
Other^d^	8 (9)	14 (11)	0.55
Second‐line	61 (58)	69 (43)	0.02
Axitinib	10 (16)	14 (20)	0.57
Everolimus	19 (31)	20 (29)	0.79
Sunitinib	7 (11)	10 (14)	0.61
Pazopanib	5 (8)	6 (9)	0.92
Nivolumab	15 (25)	11 (16)	0.22
Other^d^	5 (8)	8 (12)	0.52
Third‐line	24 (23)	32 (20)	0.37
Everolimus	5 (21)	11 (34)	0.20
Axitinib	10 (41)	8 (25)	0.27
Nivolumab	3 (13)	6 (19)	0.45
Cabozantinib	3 (13)	4 (13)	0.91
Other^d^	3 (13)	3 (9)	0.28
*Cause of death* ^b^ (%)			
Cancer‐related	53/66 (80)	95/113 (84)	0.52
Non‐cancer related	2/66 (3)	4/113 (4)	0.86
Unknown	11/66 (17)	14/113 (12)	0.43

*Note*: Patient characteristics including details of baseline demographics, disease characteristics, systemic therapy received for included cohort and cause of death, stratified by sub‐group of patients with symptomatic and asymptomatic brain metastases.

Abbreviations: IMDC: International Metastatic Renal Cell Carcinoma Database Consortium; IQR: inter‐quartile range.

^a^ Symptomatic BM was defined as a metastatic renal cell carcinoma patient identified to have one of the following symptoms at the time of brain metastasis diagnosis, as reported in the CKCis database: headache, seizure, focal neurological deficit.

^b^ For data on histology, IMDC prognostic risk and cause of death, data for all patients was not available in CKCis databases. The reported proportions are of the available data. For each variable, the denominator for total number of patients with available data is reported for each cohort.

^c^ Systemic therapy includes any systemic therapy received following diagnosis of metastatic renal cell carcinoma.

^d^ Other refers to alternative investigational treatments administered on clinical trial.

Receipt of the first‐, second‐ and third‐line therapy was lower among patients with symptomatic BM, with a statistically significant difference noted only for receipt of second‐line therapy (asymptomatic: 58% vs. symptomatic: 43%, *p* = 0.02). There were no significant differences in the type of systemic therapy received among all lines for therapy between the two groups. There was no difference in clinical trial enrollment, with 21 (20%) patients in the asymptomatic BM group and 31 (19%) in the symptomatic group enrolled in clinical trials (Table 1).

### 3.2 Characteristics of brain metastases

The majority of patients were diagnosed with multiple (i.e., >1) BM (asymptomatic: 72% versus symptomatic: 78%) with the mean number of BM (IQR) 1.72 (1, 2) and 1.78 (2), respectively (Table 2). The majority of patients received local treatment with radiation therapy (asymptomatic: 65% vs. symptomatic: 62%), with SRS being the most common approach (asymptomatic: 61% vs. symptomatic: 55%). A higher proportion of patients presenting with asymptomatic BM did not receive any local therapy (20%), as compared to patients with symptomatic BM (7%); however, this difference was not statistically significant. Table 2 summarizes the characteristics of BM in this cohort.

**TABLE 2:** Characteristics of brain metastases

Characteristic	Asymptomatic (*N* = 106)	Symptomatic (*N* = 161)	*p*‐value
Number of brain metastases (%)			0.27
Solitary	30 (28)	36 (22)	
Multiple (>1)	76 (72)	125 (78)	
Number of brain metastases (mean (IQR))	1.72 (1–2)	1.78 (2–2)	0.27
Local therapy (%)			0.87
Surgery	25 (24)	41 (25)	
Radiation therapy	69 (65)	99 (61)	
Whole brain radiotherapy	23 (33)	40 (40)	
Stereotactic radiosurgery	42 (61)	54 (55)	
Unclassified^a^	4 (6)	5 (5)	
None	21 (20)	12 (7)	

*Note*: Characteristics of brain metastases for included cohort, stratified by sub‐group of patients with symptomatic and asymptomatic brain metastases. Local therapy reported details primary therapy for brain metastases, not inclusive of any adjuvant treatments.

Abbreviations: IQR: interquartile range; SD: standard‐deviation.

^a^ Unclassified refers to radiation therapy that could not be further classified in the CKCis database.

### 3.3 Patient outcomes

Survival outcomes were evaluated from both the time of mRCC diagnosis, and from the time of BM diagnosis. The median follow‐up time for the asymptomatic and symptomatic BM group from the time of mRCC diagnosis was 36 months (IQR: 18–63 months) and 31 months (IQR: 12–55 months), respectively. The median follow‐up time for the asymptomatic and symptomatic BM group from the time of BM diagnosis was 23 months (IQR: 9–46 months) and 13 months (IQR: 5–33 months), respectively.

**FIGURE 1:**
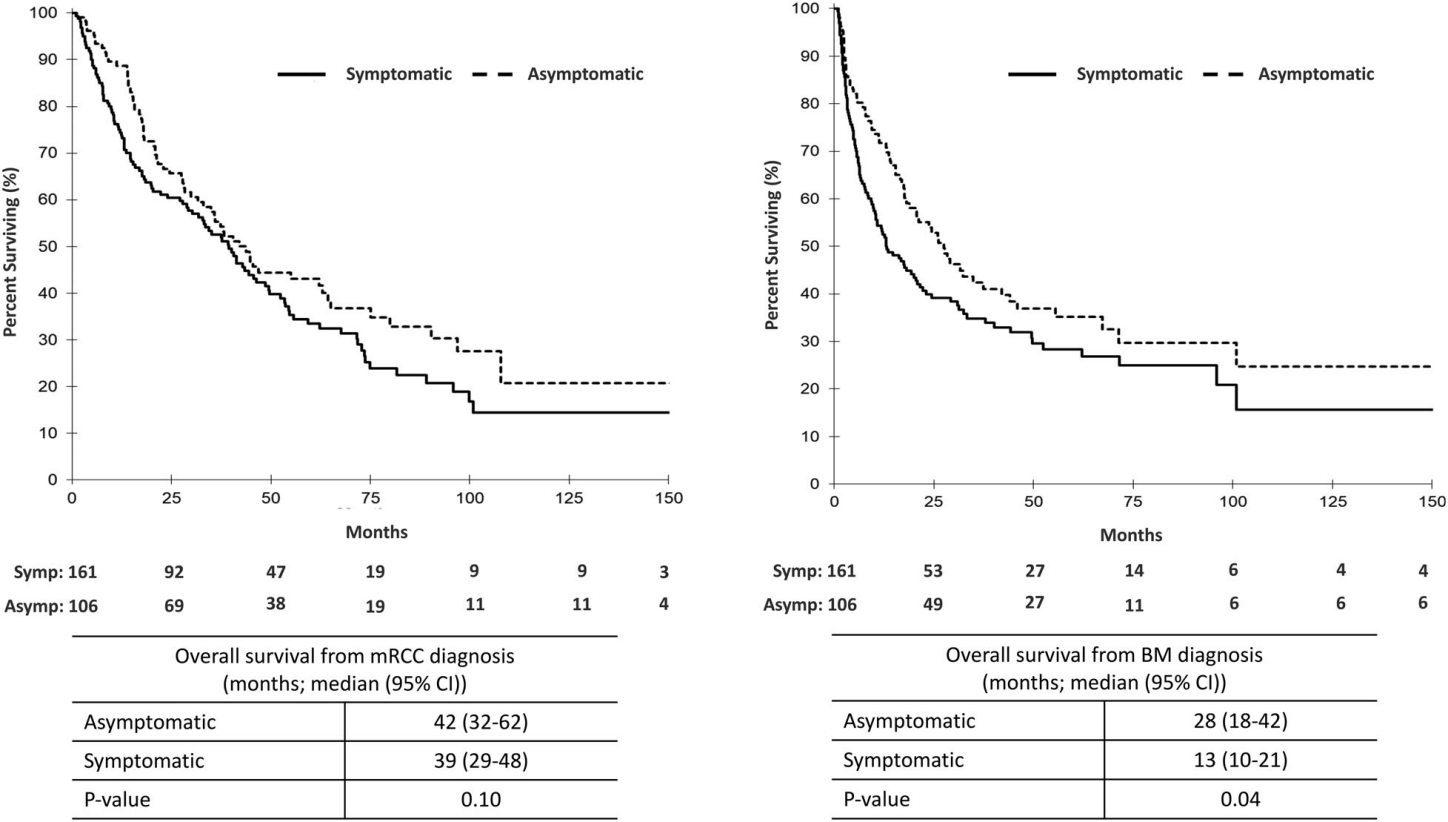
Overall survival. Kaplan–Meier OS curves for mRCC patients with asymptomatic (dashed line) and symptomatic BM (solid line). Kaplan–Meier survival curves on the left‐hand side represent OS as measured from the time of mRCC diagnosis. Kaplan–Meier survival curves on the right hand side represent OS from the time of BM diagnosis (right). Median OSs with 95% CI reported in tables below. Asym, asymptomatic; BM, brain metastases; CI, confident interval; mRCC, metastatic renal cell carcinoma; Symp, symptomatic.

From the time of mRCC diagnosis, the median OS for patients with asymptomatic BM was 42 months (95% CI: 32–62 months) and median OS for patients with symptomatic BM was 39 months (95% CI: 29–48 months) with no significant difference between the groups (*p* = 0.10). The median time from mRCC diagnosis to BM diagnosis in the asymptomatic and symptomatic BM group was 4 months (95% CI: 0–75 months) and 5 months (95% CI: 0–169 months), respectively. As calculated from the time of diagnosis of BM, a significant difference in OS was noted with a median OS of 28 months (95% CI: 18–42 months) among patients with asymptomatic BM as compared to 13 months (95% CI: 10–21 months) among patients with symptomatic BM (*p* = 0.04). Figure 1 depicts the KM survival curves.

### 3.4 Univariate analysis

Univariate analysis identified prior nephrectomy as being associated with a lower risk of mortality (HR: 0.56, 95% CI: 0.39–0.79, *p* < 0.05), while presence of symptomatic BM (HR: 1.37, 95% CI: 1.01–1.86, *p* < 0.05) and BM‐directed therapy with WBRT, as compared to SRS was associated with a higher risk of mortality (HR: 1.94, 95% CI: 1.30–2.90, *p* < 0.05). Age, sex, histology, IMDC prognostic risk classification, receipt of systemic therapy (yes versus no), presence of multiple BM and application of local therapy (yes versus no) were not found to be predictive of mortality from the time of mRCC diagnosis on univariate analysis. Supplemental Table 1 summarizes the results of the univariate analysis.

### 3.5 Multivariable analysis

Multivariable analysis revealed prior nephrectomy (HR: 0.50, 95% CI: 0.31–0.80, and *p* < 0.05) to be significantly associated with a lower risk of mortality, and BM‐directed therapy with WBRT, as compared to SRS to be significantly associated with higher risk of mortality (HR: 1.78, 95% CI: 1.17–2.73, and *p* < 0.05). Age, sex, the presence of symptomatic (as opposed to asymptomatic) BM and the presence of multiple BM were not found to be predictive of mortality from the time of mRCC diagnosis on multivariable analysis. Supplemental Table 1 summarizes the results of the multivariable analysis.

## 4 DISCUSSION

In this study we evaluated and compared the characteristics and outcomes between mRCC patients diagnosed with asymptomatic as compared to symptomatic BM. Using a national database, 9% of mRCC patients were diagnosed with BM of which 40% were asymptomatic at the time of BM diagnosis. There were no significant differences in clinical and disease characteristics between patients who presented with and without symptoms secondary to their BM. Among patients who received local therapy for their intra‐cranial disease, there was no significant differences in the type of BM‐directed treatment received. Overall survival from the time of BM diagnosis was longer among patients who presented with asymptomatic disease; however, survival outcomes from the time of mRCC diagnosis were not significantly different between the two groups.

There is a growing body of evidence describing the incidence of asymptomatic BM, with prior literature reporting estimates of asymptomatic BM in up to one‐third of mRCC patients.^2^, ^7^, ^17^ As compared to prior literature, our data revealed a higher proportion of patients diagnosed with BM in the absence of symptoms. Although the reason for this high proportion of asymptomatic BM cannot be elucidated with current data, postulated reasons for the observed high proportion of asymptomatic BM include greater access to magnetic resonance imaging in the evaluated time period which is more sensitive in identifying small lesions.^18^ In addition, the high proportion of patients identified with asymptomatic BM may be secondary to greater access to clinical trials, where intra‐cranial imaging is required to assess for clinical trial eligibility. Toward this, a recent review of patients being evaluated for clinical trial eligibility identified asymptomatic BM in 5% of mRCC patients.^17^ Similarly, Hanzley et al. reported clinical trial enrollment as the most common reason for intra‐cranial imaging in patients identified to have asymptomatic BM in their single‐center retrospective cohort study.^7^

In keeping with prior literature, concomitant lung metastases were present in the majority of patients with either asymptomatic or symptomatic BM.^7^, ^17^, ^19^ The high proportion of patients who present with metastases to both sites suggests a potential role for the presence of lung metastases to serve as a clinical indicator to screen for BM.^7^, ^17^ The efficiency of this approach, as opposed to routine screening among all mRCC patients, warrants further evaluation.

The finding of longer survival outcomes from the time of BM for patients with asymptomatic disease, as compared to symptomatic, is hypothesis generating. Although caution is required in attributing any causal relationship given the potential for a lead‐time bias, it is postulated that these results may reflect the treatment effect of timely BM‐directed therapy that has then successfully altered the disease trajectory of mRCC patients with BM resulting in an improvement in patient outcomes. The positive impact of modern radiation techniques, such as with SRS, on improving the outcomes of mRCC patients with BM as it pertains to improvement in local intra‐cranial disease control have been previously demonstrated.^1^, ^13^, ^14^, ^15^, ^20^, ^21^ As the majority of patients with asymptomatic BM received local therapy with SRS, the demonstrated improvement in survival for these patients, as compared to symptomatic BM, may be associated with an improvement in intra‐cranial disease control. Although patients with symptomatic BM received SRS to their intra‐cranial disease at similar rates, the shorter survival seen among these patients from the time of BM diagnosis may reflect less effective intra‐cranial disease control for more advanced BM. Indeed, prior literature has demonstrated poorer local disease control following SRS in the setting of more advanced intra‐cranial disease.^14^ Although there were no significant differences between the two groups with respect the number of BM, in light of the symptomatic nature of their disease, there may be other uncaptured factors that lead to higher intra‐cranial disease burden in the symptomatic cohort (i.e., tumor volume, edema, and bleeding). However, in the absence of direct data on these additional BM characteristics and the outcomes of intra‐cranial disease control following application of local therapy between the two cohorts, this remains speculative. Nevertheless, this finding highlights the importance of further prospective investigation.

The current study is one of a few that has investigated the potential role of routine intra‐cranial imaging among patients with mRCC treated with contemporary radiation techniques.^7^ Hanzley et al. also evaluated outcomes among asymptomatic versus symptomatic BM in a retrospective and single institution cohort study. Although the patient cohort in the current study, and that by Hanzley et al., was comparable with respect to clinical characteristics and use of BM‐directed therapy, Hanzley et al. described survival from the time of BM diagnosis to be longer in the cohort with symptomatic BM as compared to the asymptomatic patients.^7^ However, in their cohort, the observed survival in the symptomatic BM cohort was longer than expected for mRCC patients with BM, with a median survival that was over 5 years.

It is important to recognize the limitations of the current analysis. The retrospective design limits the ability to achieve granularity in all data variables, such as the reasons for intra‐cranial imaging among our cohort as well as, patient‐ and/or disease‐related factors that may have been considered to influence BM‐directed treatment choices. Importantly, given investigation of BM was at the discretion of the treating physicians, there may be misrepresentation of the asymptomatic BM prevalence. This lack of granularity also limited assessment of important co‐variates that have prognostic significance, such as patient performance status or complete co‐morbidities and relevant treatment‐related outcomes such as intra‐cranial and extra‐cranial disease control. Additionally, lack of data on the type of intra‐cranial imaging utilized limits assessments of the impact of imaging modality on rates of detection of BM. Thus, these data remain hypothesis‐generating only. Future investigation into the role of intra‐cranial imaging to detect asymptomatic BM for patients with mRCC is warranted.

## 5 CONCLUSION

In this study we found occult intra‐cranial disease present in 40% of patients with BM presenting with asymptomatic disease. Thus, limiting intra‐cranial imaging to mRCC patients who present with symptoms, may result in a substantial proportion of mRCC patients having a missed opportunity for timely diagnosis and BM‐directed intervention. These study results support future prospective evaluation to characterize the utility of routine intra‐cranial imaging.

## AUTHOR CONTRIBUTIONS

**Ambica Parmar:** Conceptualization (lead); data curation (lead); formal analysis (lead); writing – original draft (lead); writing – review and editing (equal). **Sunita Ghosh:** Formal analysis (equal); methodology (lead); writing – review and editing (equal). **Arjun Sahgal:** Formal analysis (equal); writing – review and editing (equal). **Aly‐Khan A. Lalani:** Writing – review and editing (equal). **Aaron R Hansen:** Writing – review and editing (equal). **Neil Reaume:** Writing – review and editing (equal). **Lori A Wood:** Writing – review and editing (equal). **Naveen S Basappa:** Writing – review and editing (equal). **Daniel Heng:** Writing – review and editing (equal). **Jeffrey Graham:** Writing – review and editing (equal). **Christian K Kollmannsberger:** Writing – review and editing (equal). **Denis Soulières:** Writing – review and editing (equal). **Rodney H Breau:** Writing – review and editing (equal). **Simon Tanguay:** Writing – review and editing (equal). **Anil Kapoor:** Writing – review and editing (equal). **Pouliot Frédéric:** Writing – review and editing (equal). **Georg Bjarnason:** Conceptualization (lead); formal analysis (lead); supervision (lead); writing – original draft (equal); writing – review and editing (lead).

## FUNDING INFORMATION

This work was supported by The Kidney Cancer Research Network of Canada (KCRNC) and the Canadian Kidney Cancer Information System (CKCis) have received unrestricted grants from Bristol‐Myers Squibb, Eisai, EMD, Serono, Glaxo‐Smith Kline, Ipsen, Pfizer, Merck, Novartis and Roche. There is no direct role or influence from this funding on this work.

## CONFLICTS OF INTEREST

**Ambica Parmar**: no disclosures. **Sunita Ghosh**: no disclosures. **Arjun Sahgal**: Honoraria: Elekta AB, Accuracy Inc, Varian, BrainLAB, Medtronic Kyphon. Consulting or advisory role: Abbvie, Merck Roche, Varian (Medical Advisory Group), Elekta (Gamma Knife Icon), ex officio Board Member to International Stereotactic Radiosurgery Society. Research funding: Elekta AB. Travel/Accomodations/Expenses: Elekta, Varian BrainLAB. **Aly‐Khan A Lalani**: Honoraria: Astellas Pharma, Bayer, Bristol‐Myers Squibb, Merck, Novartis, Pfizer, Roche/Genetech, Tersera. Consulting or advisory role: Abbvie, Astellas Pharma, Bayer, Bristol‐Myers Squibb, Eisai, Ipsen, Janssen, Merck, Pfizer, Roche/Genetech, Tersera. Research funding: Bristol‐Myers Squibb, Ipsen, Novartis, Roche. **Aaron R Hansen**: Honoraria: AstraZeneca/MedImmune, Bristol‐Myers Squibb, GlaxoSmithKline, Novartis, Merck, Pfizer. Consulting or advisory role: Boehringer Ingelheim, Boston Biomedical, Bristol‐Myers Squibb, Roche/Genetech, GlaxoSmithKline, Merck, Novartis. Research funding: Bristol‐Myers Squibb, Boehringer Ingelheim, GlaxoSmithKline, Janssen, Karyopharm Therapeutics, Merck, Novartis, Roche/Genetech. M. Neil Reaume: Honoraria: Merck, Novartis, Roche, Ipsen, AstraZeneca, Eisai. Consulting or advisory role: Pfizer, Astellas Pharma. **Lori Wood**: Research funding: Aragon Pharmaceuticals, AstraZeneca, Bristol‐Myers Squibb, Exelixis, Merck, Novartis, Pfizer, Roche Canada. **Naveen S Basappa**: Honoraria: Astellas Pharma, Eisai, Ipsen, Janssen, Merck, Pfizer. Consulting or advisory role: Astellas Pharma, AstraZeneca, Bristol‐Myers Squibb, Eisai, Ipsen, Janssen, Merck, Pfizer, Roche Canada, Bayer. Travel/Accommodations/Expenses: Eisai, Janssen. Daniel Y. C. Heng: Consulting or advisory role: Astellas Pharma, Bristol‐Myers Squibb, Eisai, Ipsen, Janssen, Merck, Novartis, Pfizer. Research funding: Bristol‐Myers Squibb, Exelixis, Ipsen, Novartis, Pfizer. **Jeffrey Graham**: no disclosures. **Christian Kollmannsberger**: Honoraria: Bristol‐Myers Squibb, Novartis, Pfizer. Consulting or advisory role: Astellas Pharma, Bristol‐Myers Squibb, Eisai, Ipsen, Janssen, Novartis, Pfizer. Travel/Accommodations/Expenses: Eisai, Novartis, Pfizer. DS: Honoraria: Merck, Pfizer, Novartis, AstraZeneca, Roche/Genetech, Ipsen, Bristol‐Myers Squibb. Consulting or advisory role: Merck, Pfizer, Ipsen. Research funding: Novartis, Pfizer, Merck, Roche/Genetech, Bristol‐Myers Squibb, Lilly. **Rodney H. Breau**: no disclosures. **Simon Tanguay**: no disclosures. **Aly‐Khan A. Lalani**: Consulting or advisory role: Amgen, Bristol‐Myers Squibb, Eisai, Ipsen, Janssen, Novartis, Pfizer. Research funding: Bristol‐Myers Squibb. **Frédéric Pouliot**: Honoraria: Sanofi, Tersera, Ferring, Merck, Bayer, Astellas, Janssen, Amgen. Consulting or advisory role: Sanofi, Tersera, Ferring, Merck, Bayer, Astellas, Janssen, Amgen. Research funding: Astellas, Merck. **Georg A. Bjarnason**: Honoraria: Bristol‐Myers Squibb, Eisai, Ipsen, Novartis, Pfizer. Consulting or advisory role: Bristol‐Myers Squibb, Eisai, Ipsen, Novartis, Pfizer. Research funding: Merck, Pfizer. Travel/Accomodations/Expenses: Novartis, Pfizer.

## ETHICS STATEMENT

The CKCis database is a National Canadian Database that has been collecting information on all RCC patients across 16 participating Canadian centers since 2011. Research ethics approval has been granted for all CKCis participating centers.

## Supporting information


**Appendix S1:** Supporting Information

## Data Availability

Data sharing is not applicable to this article as no new data were created or analyzed in this study.
