# Supplementary material for: Evaluating the impact of early identification of asymptomatic brain metastases in metastatic renal cell carcinoma
Source: Cancer Rep (Hoboken). 2022 Dec 14;6(3):e1763. doi: 10.1002/cnr2.1763 (PMC10026314; doi:10.1002/cnr2.1763)
Supplement: Supplementary file 1 — Appendix S1: Supporting Information [file CNR2-6-e1763-s001.docx]

**SUPPLEMENTAL TABLES AND FIGURES**

**Supplemental Table 1.** Univariate and multivariable analysis

| Variable | Univariate | | Multivariate | |
| --- | --- | --- | --- | --- |
|  | **HR (95% CI)** | **p-value** | **HR (95% CI)** | **p-value** |
| Age > 65 years | 1.03 (0.76,1.40) | 0.86 | 1.13 (0.73,1.73) | 0.59 |
| Male sex | 0.90 (0.64,1.26) | 0.54 | 1.20 (0.73,1.98) | 0.47 |
| Prior nephrectomy | ***0.56 (0.39,0.79)*** | ***<0.05*** | ***0.50 (0.31,0.80)*** | ***<0.05*** |
| Clear cell histology | 0.77 (0.53, 1.11) | 0.17 |  |  |
| IMDC intermediate/poor | 1.34 (0.75,2.39) | 0.32 |  |  |
| Symptomatic BM | ***1.37 (1.01,1.86)*** | ***<0.05*** | 1.31 (0.85,2.03) | 0.22 |
| Receipt of systemic therapy | 0.90 (0.60, 1.34) | 0.60 |  |  |
| Multiple BM (>1) | 1.18 (0.84,1.66) | 0.35 | 1.17 (0.66,2.06) | 0.59 |
| Local therapy (N vs. Y) | 1.30 (0.96,1.75) | 0.09 |  |  |
| WBRT vs. SRS | ***1.94 (1.30,2.90)*** | ***<0.05*** | ***1.78 (1.17,2.73)*** | ***<0.05*** |

**Table Legend.** Uni- and multi-variable analysis of clinical, disease-related and treatment-specific variables predictive of mortality from the time of mRCC diagnosis.

Abbreviations: BM: brain metastases; CI: confidence interval; HR: hazard ratio; IMDC: International Metastatic Renal Cell Carcinoma Database Consortium (IMDC); SRS: stereotactic radiosurgery; WBRT: whole brain radiotherapy.

**Supplemental Figure 1.** Sites of Metastasis

**Figure Legend.** Sites of metastatic lesions at the time of brain metastasis diagnosis for both symptomatic and asymptomatic patients
